# First Human Cases of *Leishmania (Viannia) lainsoni* Infection and a Search for the Vector Sand Flies in Ecuador

**DOI:** 10.1371/journal.pntd.0004728

**Published:** 2016-05-18

**Authors:** Hirotomo Kato, Abdon E. Bone, Tatsuyuki Mimori, Kazue Hashiguchi, Gonzalo F. Shiguango, Silvio V. Gonzales, Lenin N. Velez, Angel G. Guevara, Eduardo A. Gomez, Yoshihisa Hashiguchi

**Affiliations:** 1 Division of Medical Zoology, Department of Infection and Immunity, Jichi Medical University, Tochigi, Japan; 2 Laboratory of Parasitology, Department of Disease Control, Graduate School of Veterinary Medicine, Hokkaido University, Hokkaido, Japan; 3 Servicio Nacional de Erradicacion de la Malaria (SNEM), Ministerio de Salud Publica, Coca, Ecuador; 4 Department of Microbiology, Faculty of Life Sciences, Graduate School of Health Sciences, Kumamoto University, Kumamoto, Japan; 5 Departamento de Parasitologia y Medicina Tropical, Facultad de Ciencias Medicas, Universidad Catolica de Santiago de Guayaquil, Guayaquil, Ecuador; 6 Centro de Biomedicina, Facultad de Medicina, Universidad Central del Ecuador, Quito, Ecuador; Hitit University, Faculty of Medicine, TURKEY

## Abstract

An epidemiological study of leishmaniasis was performed in Amazonian areas of Ecuador since little information on the prevalent *Leishmania* and sand fly species responsible for the transmission is available. Of 33 clinical specimens from patients with cutaneous leishmaniasis (CL), causative parasites were identified in 25 samples based on cytochrome *b* gene analysis. As reported previously, *Leishmania (Viannia) guyanensis* and *L*. *(V*.*) braziliensis* were among the causative agents identified. In addition, *L*. *(V*.*) lainsoni*, for which infection is reported in Brazil, Bolivia, Peru, Suriname, and French Guiana, was identified in patients with CL from geographically separate areas in the Ecuadorian Amazon, corroborating the notion that *L*. *(V*.*) lainsoni* is widely distributed in South America. Sand flies were surveyed around the area where a patient with *L*. *(V*.*) lainsoni* was suspected to have been infected. However, natural infection of sand flies by *L*. *(V*.*) lainsoni* was not detected. Further extensive vector searches are necessary to define the transmission cycle of *L*. *(V*.*) lainsoni* in Ecuador.

## Author Summary

In Ecuador, leishmaniasis is endemic in Pacific coast subtropical, Amazonian, and Andean highland areas. Seven *Leishmania* species, *Leishmania* (*Leishmania*) *mexicana*, *L*. *(L*.*) amazonensis*, *L*. *(L*.*) major*-like, *Leishmania (Viannia) guyanensis*, *L*. *(V*.*) panamensis*, *L*. *(V*.*) braziliensis*, and *L*. *(V*.*) naiffi*, are reported to be associated with human cutaneous (CL) and mucocutaneous leishmaniases (MCL). Causative parasites have been studied extensively in Pacific coast and Andean areas; however, information such as prevalent *Leishmania* species and their vector sand fly species is very sparse in Amazonian areas. Giemsa-stained smears taken from patients’ skin ulcers and used for routine diagnosis of CL and Flinders Technology Associates (FTA) card-spotted samples were utilized as DNA sources, and causative parasites were identified on the basis of cytochrome *b* gene analysis. Causative parasites in 25 samples were successfully identified, and, in addition to previously reported species, *L*. *(V*.*) guyanensis* and *L*. *(V*.*) braziliensis*, *L*. *(V*.*) lainsoni* was identified from two patients living in different areas situated more than 200 km apart. Sand flies were examined in areas where one of the *L*. *(V*.*) lainsoni* infected patient was suspected to have been infected. Although 1,104 female sand flies were dissected and examined for species identification and detection of natural infection with flagellates in the gut, human-infective *Leishmania* species including *L*. *(V*.*) lainsoni* were not detected. Further extensive investigation of sand fly fauna is necessary to incriminate the vector of this parasite in Ecuador.

## Introduction

Leishmaniases are caused by infection with protozoan parasites of the genus *Leishmania* transmitted by bites of female sand flies [1, 2]. The genus *Leishmania* is further divided into two subgenera, *Leishmania* (*Leishmania*) and *Leishmania* (*Viannia*), originally distinguished by their development in the digestive tract of sand fly vectors and later confirmed by phylogenetic studies. This group of diseases is distributed worldwide, especially in tropical and subtropical areas, affecting at least 12 million people in 98 countries [2]. Approximately 20 *Leishmania* species are known to be pathogenic to humans, and the infecting species is the major determinant of clinical outcome [2]. Therefore, identification of the parasite species in endemic areas is important for both appropriate treatment and prognosis.

In Ecuador, leishmaniasis is a major public health concern and is reported in 21 of 24 provinces of the country, in Pacific coast subtropical, Amazonian, and Andean highland areas [3]. Currently, seven *Leishmania* species, *Leishmania* (*Leishmania*) *mexicana*, *L*. *(L*.*) amazonensis*, *L*. *(L*.*) major*-like, *Leishmania (Viannia) guyanensis*, *L*. *(V*.*) panamensis*, *L*. *(V*.*) braziliensis*, and *L*. *(V*.*) naiffi*, have been identified as causative agents for human cutaneous (CL) and mucocutaneous leishmaniases (MCL) [4, 5, 6]. In Pacific coast areas and Andean areas, causative parasite species have been studied extensively; *L*. *(V*.*) guyanensis*, *L*. *(V*.*) panamensis*, *L*. *(V*.*) braziliensis*, and *L*. *(L*.*) amazonensis* in Pacific areas, and *L*. *(L*.*) mexicana* as a dominant species and *L*. *(L*.*) major*-like as a minor species in Andean areas [4, 5, 7, 8, 9, 10, 11]. On the other hand, in Amazonian areas, although *L*. *(V*.*) guyanensis*, *L*. *(V*.*) braziliensis*, and recently, *L*. *(V*.*) naiffi* have been identified as causative agents for CL and MCL [4, 5, 6, 12, 13, 14], epidemiological studies on leishmaniasis have been very limited and consequently little information is available regarding prevalent parasite species as well as epidemiological situation.

Molecular biological methods are widely used for identification of *Leishmania* species using DNA extracted from clinical material of patients’ lesion, and is a powerful tool for epidemiological studies of leishmaniasis [15, 16, 17, 18]. DNA extracted from Giemsa-stained smears obtained from patients’ skin ulcers, which are used routinely for microscopic diagnosis in the laboratory to detect parasites in the lesion, have been used also as templates for detection and identification of *Leishmania* DNA although the sensitivity is low because of the limitation of the DNA source [19, 20, 21, 22]. Recently, to facilitate sample collection and DNA extraction processes, a Flinders Technology Associates (FTA) card (Whatman), a filter paper that readily lyses the spotted materials and fixes nucleic acids, was used for direct sampling of patients’ material in an epidemiological study of leishmaniasis, and its usability was reported [18, 23]. In the present study, using smear slides and FTA card-spotted samples as DNA sources, an epidemiological survey of leishmaniasis was performed in Amazonian areas where little information on the endemic *Leishmania* species and vector sand flies is available.

## Materials and Methods

### Sample collection

Clinical samples were collected from patients suspected of having CL who visited health centers in Cascales, Lago Agrio (Province of Sucumbíos), Coca, La Joya de los Sachas, and Nuevo Rocafuerte (Province of Orellana) for the diagnosis and treatment of leishmaniasis (S1 Fig). Tissue samples were taken by scraping the margins of active lesions on patients, spotting them onto FTA Classic Cards (Whatman, Newton Center, MA) and storing them at room temperature. Two-mm-diameter disks containing the sample spot were punched out from each filter paper and washed three times with FTA Purification Reagent (Whatman) and once with Tris-EDTA buffer. The disks were air-dried and directly subjected to PCR amplification. For the extraction of DNA from Giemsa-stained smears taken from patients’ skin ulcers that were used for diagnosis of CL, 30 μl of DNA extraction buffer [150 mM NaCl, 10 mM Tris-HCl (pH 8.0), 10 mM EDTA and 0.1% sodium dodecyl sulfate (SDS)] containing 100 μg/ml of proteinase K were spotted on each smear, and the tissue sample was collected into 1.5 ml microtube. The sample was incubated at 37°C overnight, heated at 95°C for 5 min, and then 0.5 μl of each sample was directly used as a template for PCR amplification.

### Sand fly collection

Sand flies were captured with mouth aspirator on protected human bait, CDC light traps, and the modified Shannon light traps [24] between 18:30 and 21:00 for 14 nights on February 2015 at mountain and forest areas around patient houses in Sucumbíos Province where a patient was suspected to have acquired an infection. Female sand flies were dissected and identified to species level based mainly on the morphology of their spermathecae [25]. Sand flies were examined under light microscopy for natural flagellate infections and positive samples were fixed individually in absolute ethanol. Ethanol-fixed specimens were individually lysed in 50 μl of DNA extraction buffer with proteinase K, and 0.5 μl of the extract was directly used as PCR templates [6, 26, 27, 28].

### Identification of *Leishmania* species

*Leishmania* species were identified by cytochrome *b* (*cyt* b) gene sequence analysis [18, 23]. PCR amplification with a pair of specific primers, L.cyt-AS (5'-GCGGAGAGRARGAAAAGGC-3') and L.cyt-AR (5'-CCACTCATAAATATACTATA-3'), was performed with 30 cycles of denaturation (95°C, 1 min), annealing (55°C, 1 min) and polymerization (72°C, 1 min) using Ampdirect Plus reagent (Shimadzu Biotech, Tsukuba, Japan). A portion of the PCR product was reamplified with L.cyt-S (5'-GGTGTAGGTTTTAGTYTAGG-3') and L.cyt-R (5'-CTACAATAAACAAATCATAATATRCAATT-3'). For some samples, *Leishmania* species were further identified by heat-shock protein 70 (*hsp70*) gene sequence analysis [29]. PCR amplification with a pair of specific primers, HSP70sen (5'-GACGGTGCCTGCCTACTTCAA-3') and HSP70ant (5'- CCGCCCATGCTCTGGTACATC-3'), was performed with 40 cycles of denaturation (95°C, 1 min), annealing (55°C, 1 min) and polymerization (72°C, 1 min) using Ampdirect Plus reagent (Shimadzu Biotech, Tsukuba, Japan). The products were cloned into the pGEM-T Easy Vector System (Promega, Madison, WI) and sequences were determined by the dideoxy chain termination method using a BigDye Terminator v3.1 Cycle Sequencing Kit (Applied Biosystems, Foster City, CA).

### Phylogenetic analysis

The *Leishmania cyt* b gene sequences were aligned with CLUSTAL W software [30] and examined using the program MEGA (Molecular Evolutionary Genetics Analysis) version 6 [31]. Phylogenetic trees were constructed by the neighbor-joining method with the distance algorithms available in the MEGA package [30]. Bootstrap values were determined with 1,000 replicates of the data sets. The database for phylogenetic analyses consisted of *cyt* b gene sequences from *L*. *(L*.*) infantum* (GenBank accession number: AB095958), *L*. *(L*.*) donovani* (AB095957), *L*. *(L*.*) major* (AB095961), *L*. *(L*.*) tropica* (AB095960), *L*. *(L*.*) amazonensis* (AB095964), *L*. *(L*.*) mexicana* (AB095963), *L*. *(V*.*) panamensis* (AB095968), *L*. *(V*.*) guyanensis* (AB095969), *L*. *(V*.*) braziliensis* (AB095966), *L*. *(V*.*) lainsoni* (AB433280), *L*. *(V*.*) naiffi* (AB433279) and *L*. *(V*.*) shawi* (AB433281).

### Ethics statement

The collection was performed by local physicians and well-trained laboratory technicians with the approval of the research ethics committee of the Graduate School of Veterinary Medicine, Hokkaido University (license number: vet26-4). Informed consent was obtained from the adult subjects and from the children’s parents or guardians, prior to collection of diagnostic materials at each health center of the Ecuadorian Ministry of Health (Provinces of Sucumbíos and Orellana). Signed consent was obtained after the explanation of the process of diagnosis and *Leishmania* species analysis at the time of routine diagnosis carried out at rural health centers, following the guidelines of the Ethics Committee of the Ministry of Health, Ecuador. The subjects studied were volunteers in routine diagnosing/screening and treatment programs promoted by the Ministry. All routine laboratory examinations were carried out free of charge, and treatment with specific drug (Glucantime) was also offered free of charge at each health center of the Ministry.

## Results

### Identification of *Leishmania* species from patient specimens

**Fig 1:**
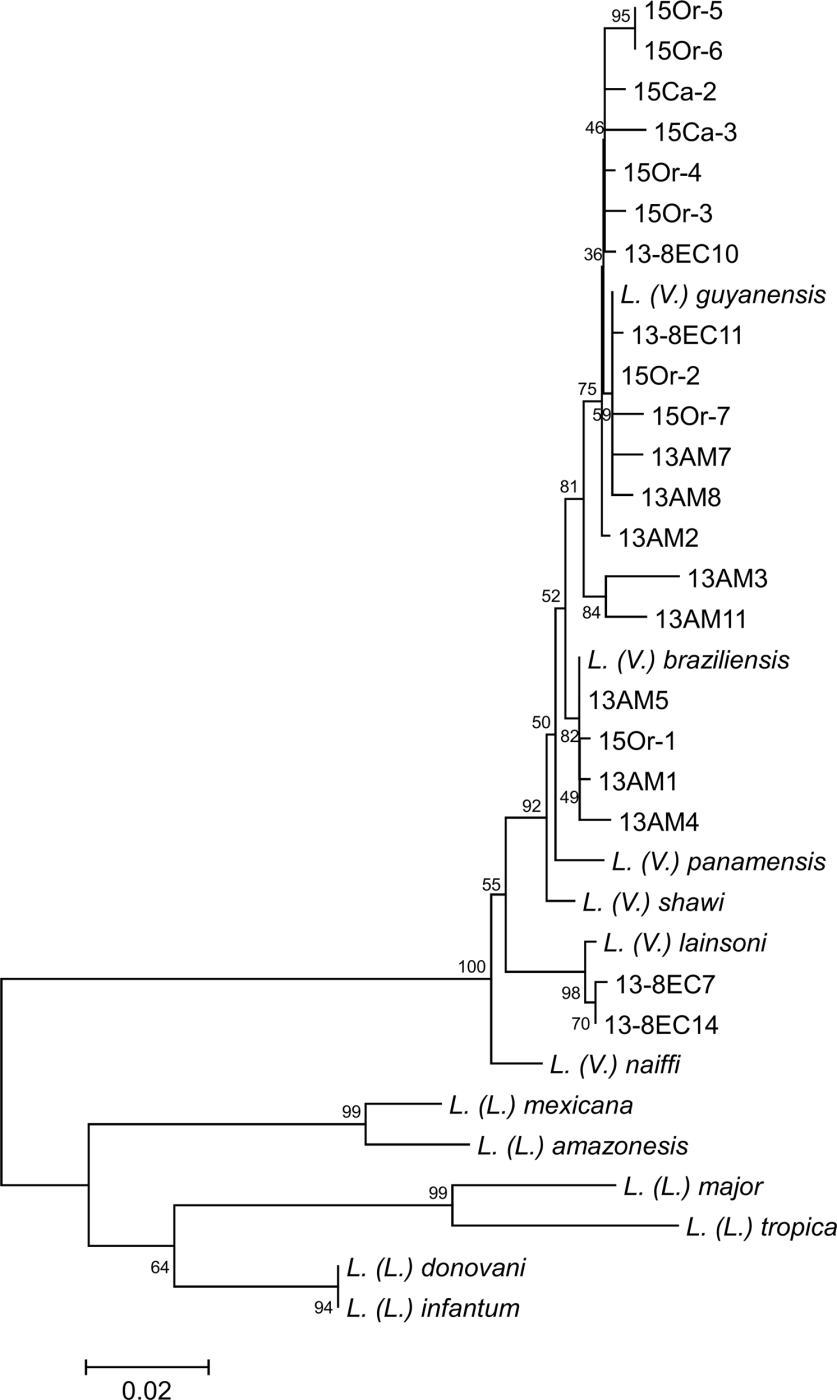
Phylogenetic tree of *cyt* b gene sequences among species. Leishmanial *cyt* b genes were amplified and sequenced from patients with cutaneous leishmaniasis (13-8EC7–13-8EC15, 13AM1–13AM11, 15Ca1–15Ca6, and 15Or1–15Or7), and a phylogenetic analysis of *cyt* b gene sequences was performed by the neighbor-joining method together with sequences from 12 *Leishmania* species. The scale bar represents 0.02% divergence. Bootstrap values are shown above or below branches.

Of 33 clinical samples, nine samples from patients in Sucumbíos Province and 24 from patients in Orellana Province were obtained (Table 1). Of these, 6 and 14 samples from Sucumbíos and Orellana patients, respectively, were Giemsa-stained smears used for routine diagnosis of CL, and others (3 and 10 samples, respectively) which were specimens spotted on FTA cards (Table 1). Patients had one to four skin lesions on their face, arms or/and legs with a diameter of 1 to 3 cm, typical of those observed on patients with CL in the area. The leishmanial *cyt* b gene was successfully amplified from two smears and three FTA card samples from Sucumbíos, and seven smear and nine FTA card samples from Orellana (Table 1), and sequences were determined. The *cyt* b gene sequences from two Sucumbíos and 13 Orellana patients had a greater degree of homology with those of *L*. *(V*.*) guyanensis* (98.1–99.8%), and *cyt* b gene sequences from two Sucumbíos and two Orellana patients had a greater degree of homology with those of *L*. *(V*.*) braziliensis* (98.6–100%). In addition, *cyt* b sequences of one specimen each from Sucumbíos and Orellana patients who have never traveled abroad showed highest homology with those of *L*. *(V*.*) lainsoni* (99.0% and 99.1%) (S2 Fig). These results were supported by a phylogenetic analysis showing that parasites from 15 patients were located in the clade of *L*. *(V*.*) guyanensis*, four specimens were in the *L*. *(V*.*) braziliensis* clade, and two samples were in the *L*. *(V*.*) lainsoni* clade (Fig 1), indicating that the causative parasite species were *L*. *(V*.*) guyanensis*, *L*. *(V*.*) braziliensis*, and *L*. *(V*.*) lainsoni*, respectively. A sample, 13-8EC7, from Sucumbíos, in which causative parasite was identified as *L*. *(V*.*) lainsoni*, was further subjected to *hsp70* gene sequence analysis. The *hsp70* gene sequence of 13-8EC7 had a greater degree of homology with those of *L*. *(V*.*) lainsoni* (99.3%), and a phylogenetic analysis supported the result (S3 Fig). Nucleotide sequence data reported are available in the DDBJ/EMBL/GenBank databases under the accession numbers LC055616, LC055617, and LC055622-LC055638.

**Table 1:** Identification of *Leishmania* species from patient specimens.

Province	Sample code	DNA source	PCR	Identification
Sucumbios	15Ca1	Smear	-	
	15Ca2	Smear	+	*L*. *(V*.*) guyanensis*
	15Ca3	Smear	+	*L*. *(V*.*) guyanensis*
	15Ca4	Smear	-	
	15Ca5	Smear	-	
	15Ca6	Smear	-	
	13-8EC7	FTA card	+	*L*. *(V*.*) lainsoni*
	13AM1	FTA card	+	*L*. *(V*.*) braziliensis*
	13AM4	FTA card	+	*L*. *(V*.*) braziliensis*
Orellana	13-8EC9	Smear	-	
	13-8EC10	Smear	+	*L*. *(V*.*) guyanensis*
	13-8EC11	Smear	+	*L*. *(V*.*) guyanensis*
	13-8EC12	Smear	-	
	13-8EC13	Smear	-	
	13-8EC14	Smear	+	*L*. *(V*.*) lainsoni*
	13-8EC15	Smear	-	
	13AM5	Smear	+	*L*. *(V*.*) braziliensis*
	13AM6	Smear	-	
	13AM7	Smear	+	*L*. *(V*.*) guyanensis*
	13AM8	Smear	+	*L*. *(V*.*) guyanensis*
	13AM9	Smear	-	
	13AM10	Smear	-	
	13AM11	Smear	+	*L*. *(V*.*) guyanensis*
	13-8EC8	FTA card	-	
	13AM2	FTA card	+	*L*. *(V*.*) guyanensis*
	13AM3	FTA card	+	*L*. *(V*.*) guyanensis*
	15Or1	FTA card	+	*L*. *(V*.*) braziliensis*
	15Or2	FTA card	+	*L*. *(V*.*) guyanensis*
	15Or3	FTA card	+	*L*. *(V*.*) guyanensis*
	15Or4	FTA card	+	*L*. *(V*.*) guyanensis*
	15Or5	FTA card	+	*L*. *(V*.*) guyanensis*
	15Or6	FTA card	+	*L*. *(V*.*) guyanensis*
	15Or7	FTA card	+	*L*. *(V*.*) guyanensis*

### Sand fly collection and dissection

**Fig 2:**
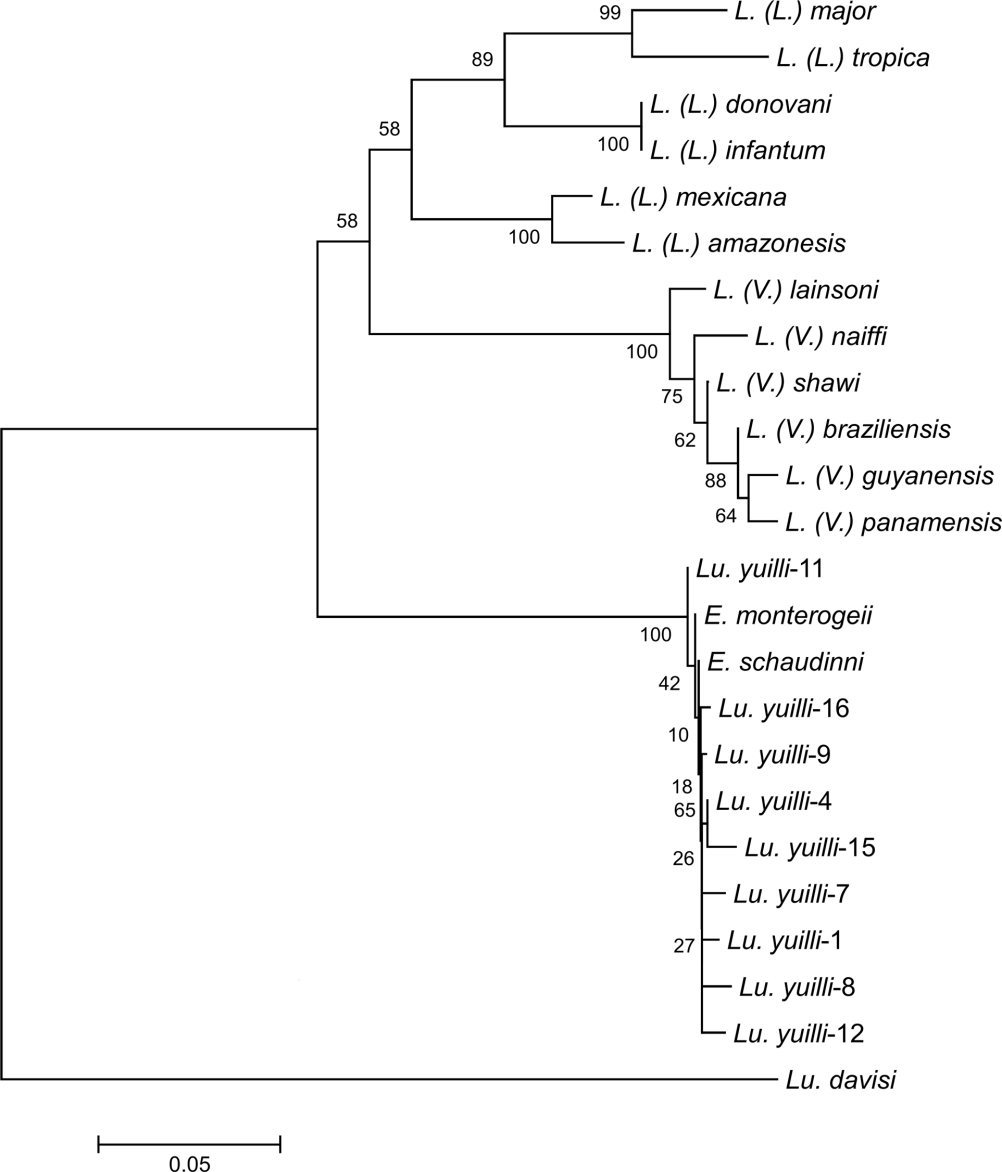
Phylogenetic tree of *cyt* b gene sequences among species. Leishmanial *cyt* b genes were amplified and sequenced from flagellates-positive sand flies, *Lutzomyia (Lu*.*) yuilli yuilli* (1, 4, 7, 8, 9, 11, 12, 15, and 16) and *Lu*. *davisi*, and a phylogenetic analysis of *cyt* b gene sequences was performed by the neighbor-joining method together with sequences from 12 *Leishmania* and 2 *Endotrypanum* species. The scale bar represents 0.05% divergence. Bootstrap values are shown above or below branches.

A total of 1,104 female sand flies were captured and dissected. Of these, 732 were captured on protected human bait, and 372 flies with light traps (CDC light trap and the modified Shannon trap) (Table 2). Four species, *Lu*. *yuilli yuilli* (71.7%), *Lu*. *tortura* (12.3%), *Lu*. *davisi* (7.3%), and *Lu*. *napoensis* (5.0%) accounted for 96.3% of the sand flies (Table 2). Among these, *Lu*. *napoensis* was captured only by light traps. In addition to these four species, *Lu*. *sherlocki*, *Lu*. *trapidoi*, *Lu*. *gomezi*, *Lu marinkelli*, *Lu dysponeta*, *Lu camposi*, *Lu*. *robusta*, *Lu hirusta hirusta*, *Lu micropyga*, and five unidentified species were captured (Table 2). Natural flagellate infections were observed in the hindguts of 14 *Lu*. *yuilli yuilli* (1.8%), one *Lu*. *davisi* (1.2%), and of the only collected specimen of *Lu*. *camposi* (Table 2). Genomic DNAs were extracted from dissected sand flies infected with flagellates, and parasite *cyt* b genes were amplified. The *cyt* b gene fragments were successfully obtained from nine of the 14 positive *Lu*. *yuilli yuilli* and from *Lu*. *davisi*. The unsuccessful amplification of parasite *cyt* b genes from the other five *Lu*. *yuilli yuilli* and *Lu*. *camposi* was attributed to the very small number of parasites present in the gut. The *cyt* b gene sequences of parasites from the 10 flagellate-positive sand flies were analyzed and compared to those of related parasite species. The *cyt* b gene sequences from the nine *Lu*. *yuilli yuilli* showed only 86.6–88.6% homology with those of the *Leishmania* species, and 99.1–99.8% homology with those of *Endotrypanum* species, flagellate parasites of non-human animals transmitted by sand flies [32, 33], indicating that the flagellate infections in *Lu*. *yuilli yuilli* were *Endotrypanum*. On the other hand, the sequences of the parasite from *Lu*. *davisi* had only 81.2–82.3% homology with those of *Leishmania* and *Endotrypanum* species, indicating that the flagellate is neither *Leishmania* nor *Endotrypanum*. These results were supported by a phylogenetic analysis showing that nine flagellates from *Lu*. *yuilli yuilli* were located in the clade of *Endotrypanum* species, while the one from *Lu*. *davisi* was distant from *Leishmania* and *Endotrypanum* species (Fig 2). We suspect that the flagellates observed in *Lu*. *davisi* may belong to the genus *Trypanosoma* since some sand fly species are reported to transmit *Trypanosoma* species [28, 34, 35]. The vector species of *L*. *(V*.*) lainsoni* in Ecuador could not be identified in this study.

**Table 2:** Identification of sand fly species and detection of flagellates within individual sand flies by microscopic examinations.

	Human bait		Light trap	
*Lu*. *yuilli yuilli*	540	(10*)	251	(4)
*Lu*. *tortura*	111		25	
*Lu*. *davisi*	56		25	(1)
*Lu*. *sherlocki*	12		5	
*Lu*. *trapidoi*	4		0	
*Lu*. *gomezi*	2		0	
*Lu*. *marinkelli*	2		1	
*Lu*. *robusta*	1		0	
*Lu*. *napoensis*	0		55	
*Lu*. *dysponeta*	0		4	
*Lu*. *camposi*	0		1	(1)
*Lu*. *hirusta hirusta*	0		1	
*Lu*. *micropyga*	0		1	
Others	4	[2 spp.]	3	[3 spp.]
Total	732		372	

*flagellate-positive sand flies

## Discussion

An epidemiological study of leishmaniasis was conducted in Amazonian areas of Ecuador where very little information is available on prevalent parasite species or sand fly species associated with their transmission. In addition to *L*. *(V*.*) guyanensis* and *L*. *(V*.*) braziliensis* infections, the first cases of CL caused by *L*. *(V*.*) lainsoni* infection in Ecuador were identified. A search for the vector sand fly in the area where one *L*. *(V*.*) lainsoni* infected patient presumably contracted the disease produced no positive flies, thus the vector remains unknown.

Leishmaniasis is widely distributed in Pacific coast subtropical climate areas, Andean highland areas, and Amazonian tropical areas in Ecuador [4, 5]. To date, most clinical and parasitological studies of leishmaniasis have been reported from Pacific coast and Andean highland areas [4, 5, 7, 8, 9, 10, 11], and little information is available on endemic parasite and sand fly species in Amazonian areas, mainly because of difficulty in gaining access [4, 5, 6, 12, 13, 14]. In this study, Giemsa-stained smears used for routine diagnosis of CL were utilized as a DNA source in addition to FTA card-collected samples. The detection ratio was lower in DNA samples from smear slides when compared to FTA card collection (2/6 vs. 3/3 in Sucumbíos and 7/14 vs. 9/10 in Orellana), reflecting the amount of DNA recoverable and the condition of the smear slides. The thin smear samples were methanol-fixed, Giemsa-stained, and then examined under oil immersion. Thus, part of specimens and DNA may be lost and damaged when the immersion oil was wiped from the slide. Nevertheless, stored smear slides, which will not be used for any further purpose than diagnosis by microscopic examination, can be useful for identification of *Leishmania* species in endemic areas where sample collection for epidemiological study is difficult.

In this study, *L*. *(V*.*) guyanensis* and *L*. *(V*.*) braziliensis* were identified as causative agents in Amazonian areas as reported previously [4, 5, 6, 12, 13, 14]. In addition, *L*. *(V*.*) lainsoni* is implicated as a causative agent of CL in Ecuador for the first time. *Leishmania (V*.*) lainsoni* was originally identified from patients in Brazilian Amazon [36]. It causes CL with characteristic lesions similar to those caused by other *Leishmania (Viannia)* species: small ulcers or small self-limiting nodules [37]. The parasite was subsequently identified from patients in Sub-Andean and Amazonian areas of Peru [18, 38, 39, 40], in subtropical climate areas and Sub Andean areas of Bolivia [41, 42], Suriname [43], and French Giana [44]. Two *L*. *(V*.*) lainsoni*-infected patients were found during this study. One was from the northern part of Ecuadorian Amazon (Sucumbios) near the border with Colombia, and the other was from the eastern frontier with Peru (Orellana), more than 200 km away. These findings suggest that *L*. *(V*.*) laisoni* is widely distributed in South America.

Natural infections with *L*. *(V*.*) lainsoni* in sand flies have been detected in *Lu*. *ubiquitalis* in Brazil [45], *Lu*. *nuneztovari anglesi* in Bolivia [42], and *Lu*. *auraensis* in Peru [46]. Microscopic examinations of sand flies collected in this study revealed no natural infection with *L*. *(V*.*) lainsoni*, and none of the above three *Lutzomyia* species were collected. Of the three dominant human-biting species in our collections, infection with *Endotrypanum* species, flagellate parasites of sloths, which are non-pathogenic to humans [32, 33], were detected in the most dominant species, *Lu*. *yuilli yuilli*, as reported in another Amazonian area of Ecuador [6]. Although natural infection was not detected in this study, the next dominant species, *Lu*. *tortura*, was already incriminated as a vector species of *L*. *(V*.*) naiffi* in Ecuadorian Amazon [6, 13]. Furthermore, *Lu*. *davisi* was reported to transmit *L*. *(V*.*) naiffi* in Brazil [47] and *L*. *(V*.*) braziliensis* in Peru [46]. In this study, natural infection of *Lu*. *davisi* by flagellates was observed. However, the parasite was neither *Leishmania* nor *Endotrypanum*. To identify the vector of *L*. *(V*.*) lainsoni*, further research is necessary to understand the natural transmission cycle of this parasite in Ecuador.

Our finding of the first human cases of CL caused by *L*. *(V*.*) lainsoni* infection in two separate areas of Ecuador suggests that this parasite is widely distributed in South America. Extensive countrywide surveillance is necessary to gain a proper understand of the status of *L*. *(V*.*) lainsoni*, as well as the sand flies responsible for its transmission in Ecuador.

## Supporting Information

S1 Fig Map of sample collection sites in Ecuador. 1. Cascales, 2. Lago Agrio (Province of Sucumbíos), 3. Coca, 4. La Joya de los Sachas, and 5. Nuevo Rocafuerte (Province of Orellana).
(TIF)

S2 Fig Alignment of cytochrome *b* gene sequences of *Leishmania (Viannia) lainsoni*. Cytochrome *b* gene sequences obtained from Sucumbíos (13-8EC7) and Orellana (13-8EC14) patients were aligned with that of *L*. *(V*.*) lainsoni* (AB433280). Black-shaded sequences represent identical nucleotides.
(TIF)

S3 Fig Phylogenetic tree of *hsp70* gene sequences among species. Leishmanial *hsp70* gene was amplified and sequenced from a patient with cutaneous leishmaniasis (13-8EC7), and a phylogenetic analysis of *hsp70* gene sequences was performed by the neighbor-joining method together with sequences from 9 *Leishmania* species. The scale bar represents 0.005% divergence. Bootstrap values are shown above or below branches. The database for phylogenetic analyses consisted of *hsp70* gene sequences from *L*. *(L*.*) tropica* (GenBank accession number: FN395026), *L*. *(L*.*) major* (XM_001684512), *L*. *(L*.*) donovani* (X52314), *L*. *(L*.*) infantum* (XM_001470287), *L*. *(L*.*) mexicana* (EU599091), *L*. *(V*.*) braziliensis* (XM_001566275), *L*. *(V*.*) guyanensis* (EU599093), *L*. *(V*.*) naiffi* (FN395056), and *L*. *(V*.*) lainsoni* (FN395047 and FN395048).
(TIF)
