# Supplementary figures and images for: First Human Cases of Leishmania (Viannia) lainsoni Infection and a Search for the Vector Sand Flies in Ecuador
Source: PLoS Negl Trop Dis. 2016 May 18;10(5):e0004728. doi: 10.1371/journal.pntd.0004728 (PMC4871579; doi:10.1371/journal.pntd.0004728)

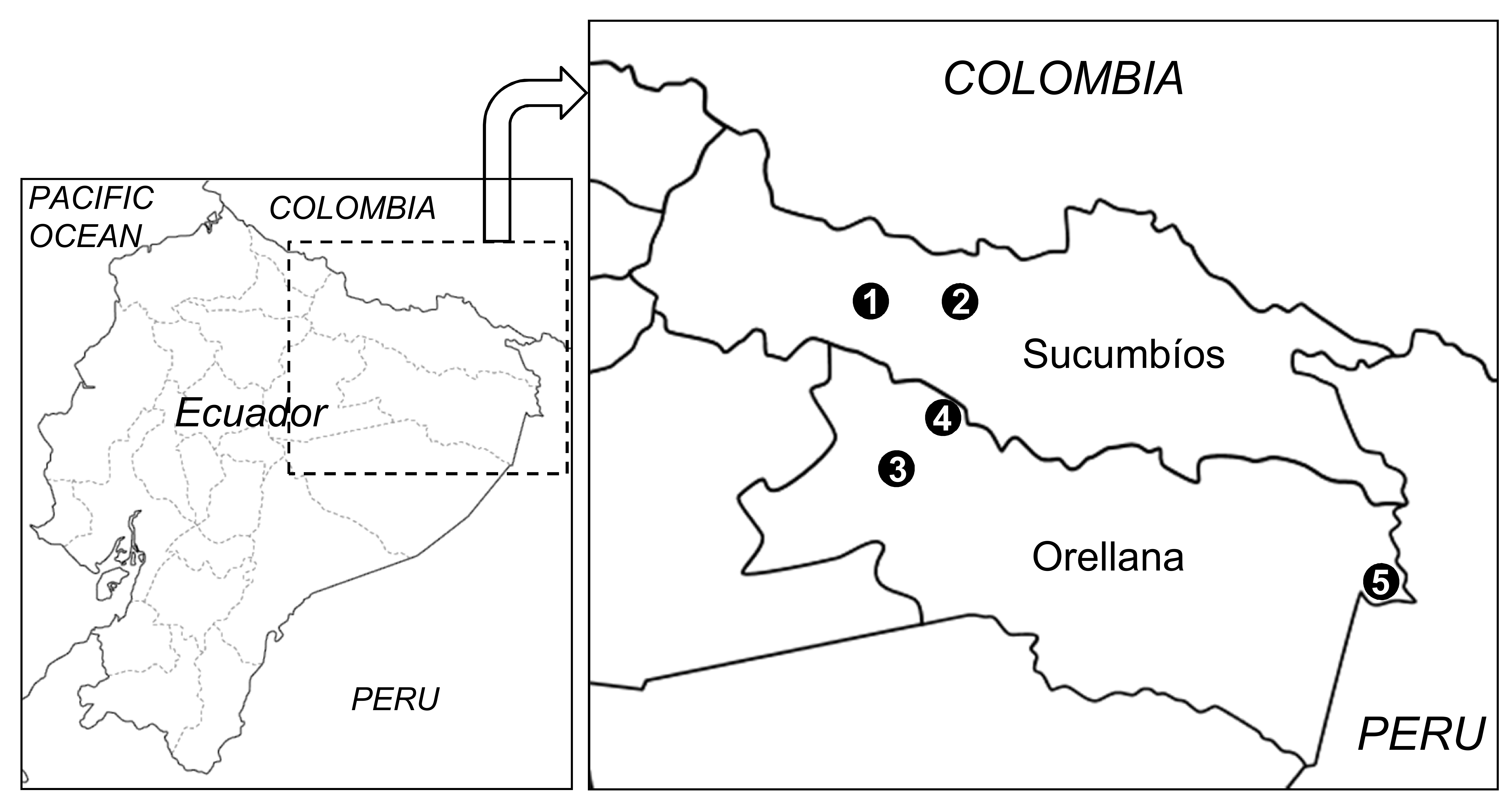

Supplement: S1 Fig — 1. Cascales, 2. Lago Agrio (Province of Sucumbíos), 3. Coca, 4. La Joya de los Sachas, and 5. Nuevo Rocafuerte (Province of Orellana). (TIF) [file pntd.0004728.s001.tif]

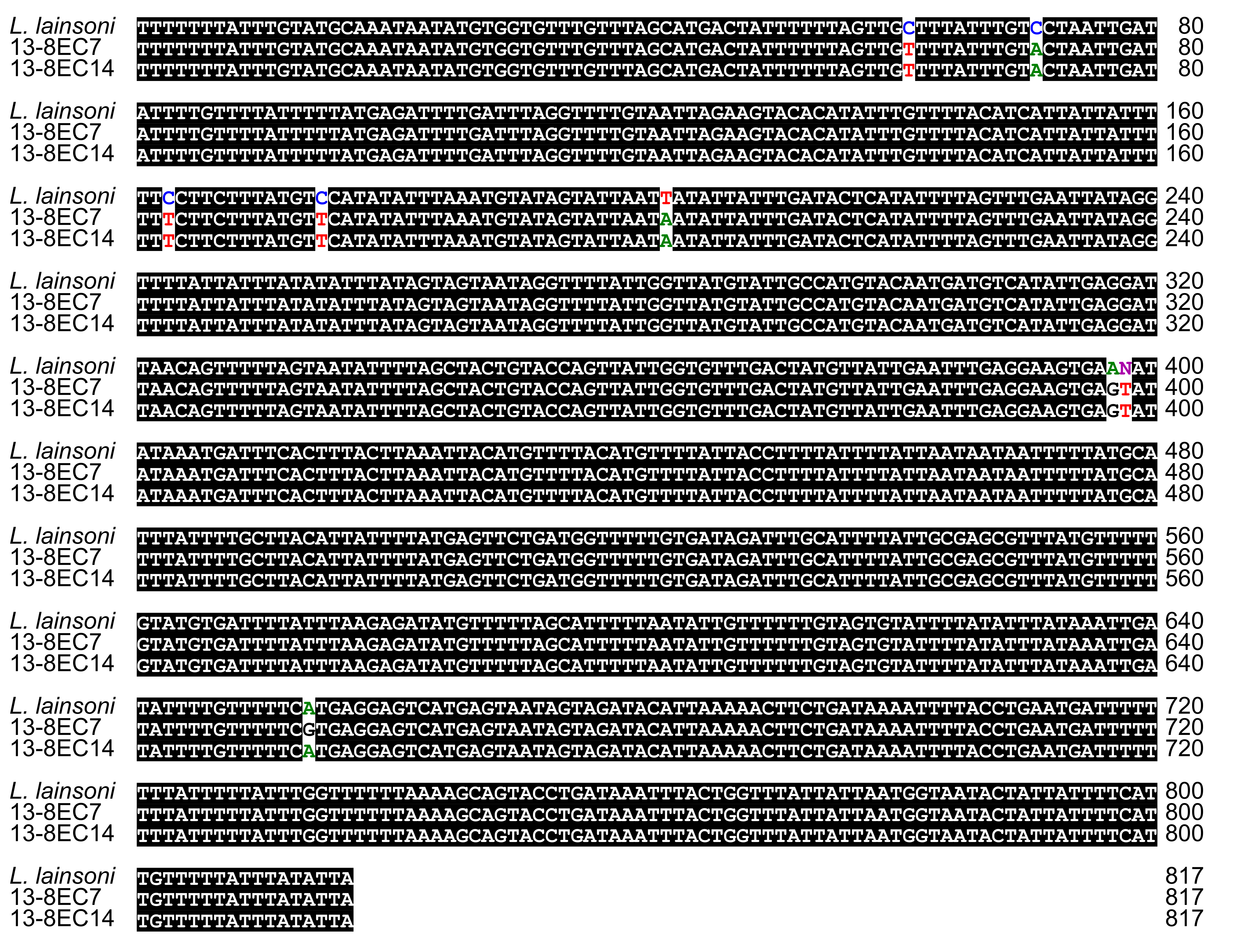

Supplement: S2 Fig — Cytochrome b gene sequences obtained from Sucumbíos (13-8EC7) and Orellana (13-8EC14) patients were aligned with that of L. (V.) lainsoni (AB433280). Black-shaded sequences represent identical nucleotides. (TIF) [file pntd.0004728.s002.tif]

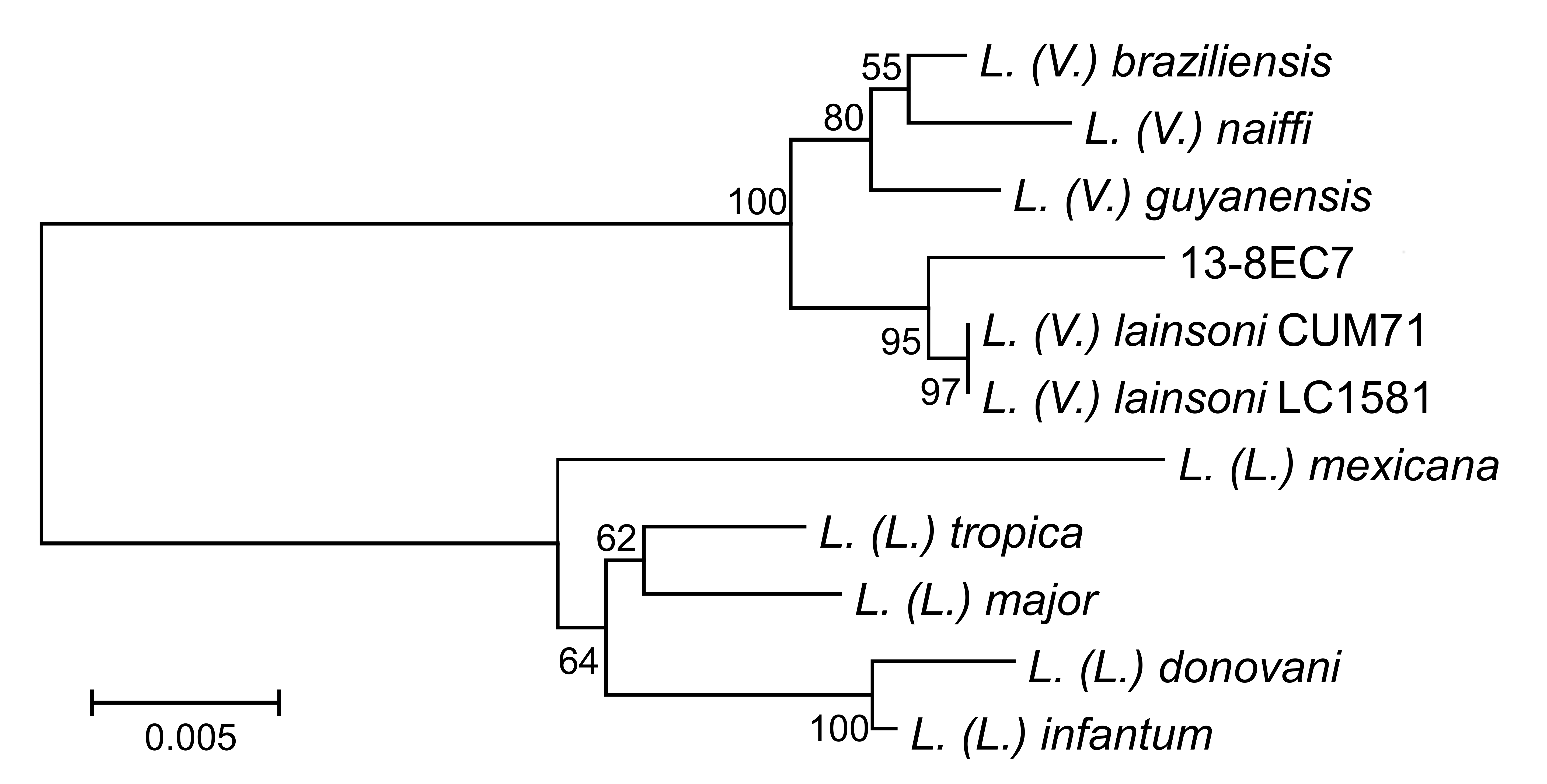

Supplement: S3 Fig — Leishmanial hsp70 gene was amplified and sequenced from a patient with cutaneous leishmaniasis (13-8EC7), and a phylogenetic analysis of hsp70 gene sequences was performed by the neighbor-joining method together with sequences from 9 Leishmania species. The scale bar represents 0.005% divergence. Bootstrap values are shown above or below branches. The database for phylogenetic analyses consisted of hsp70 gene sequences from L. (L.) tropica (GenBank accession number: FN395026), L. (L.) major (XM_001684512), L. (L.) donovani (X52314), L. (L.) infantum (XM_001470287), L. (L.) mexicana (EU599091), L. (V.) braziliensis (XM_001566275), L. (V.) guyanensis (EU599093), L. (V.) naiffi (FN395056), and L. (V.) lainsoni (FN395047 and FN395048). (TIF) [file pntd.0004728.s003.tif]
